# 1q/19p co-polysomy predicts longer survival in patients with astrocytic gliomas

**DOI:** 10.18632/oncotarget.17947

**Published:** 2017-05-16

**Authors:** Wei Zeng, Xiaohui Ren, Yong Cui, Haihui Jiang, Xiuru Zhang, Song Lin

**Affiliations:** ^1^ Department of Neurosurgery, Beijing Tiantan Hospital, Capital Medical University, Beijing, China; ^2^ Department of Pathology, Beijing Tiantan Hospital, Capital Medical University, Beijing, China; ^3^ China National Clinical Research Center for Neurological Diseases, Beijing, China; ^4^ Beijing Institute for Brain Disorders and Beijing Key Laboratory of Brain Tumor, Beijing, China

**Keywords:** 1q/19p co-polysomy, single polysomy, astrocytic gliomas, overall survival, progression-free survival

## Abstract

Recently, we reported that 1q/19p co-polysomy predicted poor prognosis in oligodendroglial tumors. In this study, we aimed to retrospectively analyze the prognostic significance of 1q/19p polysomy in two large cohorts of astrocytic gliomas classified by the 2007 and 2016 WHO classification of tumors of the central nervous system. 1q/19p polysomy was detected using the FISH method, and factors that correlated with polysomy were analyzed by logistic regression. Survival analysis was used to identify independent prognostic factors correlated with survival. In the WHO_2007_ astrocytic glioma cohort (N=421), co-polysomy was associated with a younger age, whereas single polysomy was associated with higher tumor grades and a higher Ki-67 index (*P*<0.05). Co-polysomy predicted longer survival, and single polysomy predicted shorter survival (*P*<0.05). In multivariate analysis, co-polysomy maintained an independent prognostic impact on survival (*P*=0.001) after adjustment for age, KPS, grade, removal degree, tumor size, Ki-67 index, and IDH1/2. In the WHO_2016_ cohort (N=572), we validated the prognostic merit of co-polysomy after adjusting for related factors. In conclusion, 1q/19p co-polysomy added prognostic information in cases of astrocytic glioma and could be used for molecular stratification of this disease.

## INTRODUCTION

Gliomas are the most common intracranial malignant tumor and often have a poor prognoses [1, 2]. Recently, molecular biomarkers including 1p/19q co-deletion, BRAF mutation, IDH1/2 mutation, and TERT mutation have been widely used for glioma diagnosis, treatment, and prognosis prediction [3]. The 1p/19q co-deletion is a unique genetic characteristic of oligodendroglial tumors [4, 5]. This co-deletion predicts enhanced sensitivity to radiotherapy and chemotherapy and is included in the 2016 World Health Organization (WHO) Classification of Tumors of the Central Nervous System [6]. In the detection of 1p/19q deletions by fluorescence *in situ* hybridization (FISH), polysomy of 1q and 19p was frequently encountered, which indicated two or more 1q/19p reference signals in the tumor nuclei. Some studies have found that 1q/19p co-polysomy is associated with poor outcomes in oligodendrogliomas [7–10]. However, the frequency of 1q/19p co-polysomy and its prognostic significance are still unknown in astrocytic tumors. Therefore, this study aimed to analyze the prognostic significance of 1q/19p polysomy in the WHO_2007_ (N=421) and WHO_2016_ (N=572) classified cohorts, respectively.

## RESULTS

### Overall characteristics of the WHO_2007_ cohort

The study cohort consisted of 421 patients with astrocytic glioma. The clinical and molecular details of all of the patients are summarized in Table 1. The patient ages ranged from 14 to 72 years with a mean of 43 ± 13 years. Two hundred fifty-three (60.1%) were male, and 168 (39.9%) were female. The median preoperative KPS score was 90 (IQR 10-90). Tumor sizes ranged from 1.2 to 11.0 cm with a mean size of 5.2±1.8 cm. Two hundred seventy-five patients (65.3%) received gross total resection (GTR) of the tumor, and 146 patients (34.7%) received non-GTR resection. Two hundred sixty-seven patients (63.4%) received postoperative chemotherapy, and 298 patients (70.8%) received postoperative radiotherapy.

**Table 1:** Baseline characteristics for WHO_2007_ cohort (N=421)

Clinical characteristics	No. of patients (%)			
	WHO II	WHO III	WHO IV	All
Gender				
Male	95 (59.7%)	52 (63.4%)	106 (58.9%)	253 (60.1%)
Female	64 (40.3%)	30 (36.6%)	74 (41.1%)	168 (39.9%)
Age (yrs)				
Mean+SD	39±11	39±12	50±12	43±13
Range	16-64	14-72	14-71	14-72
Tumor size (cm)				
Mean	4.9±2.0	5.5±1.9	5.5±1.5	5.2±1.8
Range	1.2-11.0	2.0-10.0	2.0-10.0	1.2-11.0
KPS score				
Median	90	90	80	90
Range	50-90	30-90	10-90	10-90
N/A	74	31	20	125
Resection				
GTR	91 (57.2%)	51 (62.2%)	133 (73.9%)	275 (65.3%)
Non-GTR	68 (42.8%)	31 (37.8%)	47 (26.1%)	146 (34.7%)
Chemotherapy				
Yes	59 (37.1%)	56 (68.3%)	152 (84.4%)	267 (63.4%)
No	87 (54.7%)	16 (19.5%)	21 (11.7%)	124 (29.5%)
N/A	13 (8.2%)	10 (12.2%)	7 (3.9%)	30 (7.1%)
Radiotherapy				
Yes	77 (48.4%)	59 (72.0%)	162 (90.0%)	298 (70.8%)
No	69 (43.4%)	12 (14.6%)	9 (5.0%)	90 (21.4%)
N/A	13 (8.2%)	11 (13.4%)	9 (5.0%)	33 (7.8%)
1q/19p polysomy				
1q single polysomy	5 (3.1%)	4 (4.9%)	9 (5.0%)	18 (4.3%)
19p single polysomy	4 (2.5%)	9 (10.9%)	27 (15.0%)	40 (9.5%)
Co-polysomy	33 (20.8%)	24 (29.3%)	28 (15.6%)	85 (20.2%)
No polysomy	117 (73.6%)	45 (54.9%)	116 (64.4%)	278 (66.0%)
IDH1/2 mutation				
Yes	65 (40.9%)	30 (36.6%)	31 (17.2%)	126 (29.9%)
No	53 (33.3%)	39 (47.6%)	111 (61.7%)	203 (48.2%)
N/A	41 (25.8%)	13 (15.9%)	38 (21.1%)	92 (21.9%)
Follow-up				
Progression	62/155 (40.0%)	44/77 (57.1%)	137/173 (79.2%)	243/405* (60.0%)
Mean PFS (mos)	48.0 (95%CI 34.9-61.1)	21.0 (95%CI 13.5-28.6)	10.0 (95%CI 8.5-11.5)	19.5 (95%CI 15.7-23.3)
Dead	32/155 (20.6%)	29/78 (37.2%)	103/180 (57.2%)	164/413* (39.7%)
Mean OS (mos)	N/A	30.0 (95%CI 20.2-39.7)	21.5 (95%CI 17.5-25.5)	45.0 (95%CI 33.6-56.4)

* PFS was not available in 16 cases and OS was not available in 8 cases. N/A= not available. GTR=gross-total resection.

1q single polysomy was found in 18 (4.3%) cases, 19p single polysomy in 40 (9.5%) cases and 1q/19p co-polysomy in 85 (20.2%) cases.

At the last follow-up, 243 of 405 patients (60.0%) experienced tumor progression, and 164 of 413 patients (39.7%) were dead. The median PFS and OS were 19.5 (95% CI 15.7-23.3) months and 45.0 (95% CI 33.6-56.4) months, respectively.

### Factors correlated with 1q/19p co-polysomy and single polysomy

The factors associated with 1q/19p co-polysomy or single polysomy was analyzed by Chi-square test, including patient age, gender, tumor size, KPS score, resection degree, chemotherapy, radiotherapy, tumor grade, IDH1/2 mutation and Ki-67 proliferation index, as shown in Table 2.

**Table 2:** clinical factors in association with polysomy in WHO_2007_ cohort (N=421)

Clinical factors		Frequency co-polysomy		*P* value	Frequency of single polysomy		*P* value
		Yes	No		Yes	No	
Gender	Male	18.6% (47/253)	81.4%(206/253)	0.323	14.6%(37/253)	85.4% (216/253)	0.567
	Female	22.6% (38/168)	77.4%(130/168)		12.5%(21/168)	87.5% (147/168)	
Age (yrs)	≤40	28.4% (46/162)	71.6%(116/162)	**0.001**	9.3% (15/162)	90.7% (147/162)	**0.041**
	>40	15.1% (39/259)	84.9%(220/259)		16.6% (43/259)	83.4% (216/259)	
Tumor size	≥6 cm	18.5% (31/168)	81.5% (137/168)	0.536	17.3%(29/168)	82.7% (139/168)	0.112
	<6 cm	21.3% (54/253)	78.7% (199/253)		11.5% (29/253)	88.5% (224/253)	
KPS score*	≥70	20.7% (49/237)	79.3% (188/237)	0.858	13.1% (31/237)	86.9% (206/237)	0.527
	<70	22.0% (13/59)	78.0% (46/59)		16.9% (10/59)	83.1% (49/59)	
Resection degree	GTR	20.4% (56/275)	79.6% (219/275)	0.903	13.1% (36/275)	86.9% (239/275)	0.656
	Non-GTR	19.9% (29/146)	80.1% (117/146)		15.1% (22/146)	84.9% (124/146)	
Chemotherapy	Yes	22.1% (59/267)	77.9% (208/267)	0.135	15.4% (41/267)	84.6% (226/267)	0.053
	No	15.3% (19/124)	84.7% (105/124)		8.1% (10/124)	91.9% (114/124)	
Radiotherapy*	Yes	20.5% (61/298)	79.5% (237/298)	0.881	15.1% (45/298)	84.9% (253/298)	0.049
	No	18.9% (17/90)	81.1% (73/90)		6.7% (6/90)	93.3% (84/90)	
Tumor grade	WHO II	20.8% (33/159)	79.2% (126/159)	**0.036**	5.7% (9/159)	94.3% (150/159)	**0.001**
	WHO III	29.3% (24/82)	70.7% (58/82)		15.9% (13/82)	84.1% (69/82)	
	WHO IV	15.6% (28/180)	84.4% (152/180)		20.0% (36/180)	80.0% (144/180)	
IDH1/2 mutation*	Yes	18.3% (23/126)	81.7% (103/126)	0.224	12.7% (16/126)	87.3% (110/126)	0.378
	No	13.3% (27/203)	86.7% (176/203)		16.3% (33/203)	83.7% (170/203)	
Ki-67 index*	<20%	20.7% (63/304)	79.3% 241/304)	0.646	10.9% (33/304)	89.1% (271/304)	**0.002**
	≥20%	18.1% (15/83)	81.9% (68/83)		25.3% (21/83)	74.7% (62/83)	

* KPS was available in 296 cases. Radiotherapy was available in 388 cases. IDH1/2 mutation was available in 329 cases. Ki-67 was available in 387 cases. GTR=gross-total resection.

Univariate analysis revealed that age (*P*=0.001), and tumor grade (*P*=0.036) were correlated with 1q/19p co-polysomy. Logistic regression analysis confirmed age ≤40 (OR 2.237, 95%CI 1.381-3.623, *P*=0.001) as an independent factor correlated with 1q/19p co-polysomy (Table 3).

**Table 3:** Logistic regression of factors correlated with polysomy in WHO_2007_ cohort

Clinical Factors	OR (95% CI)	*P* value
Factors correlated with co-polysomy		
Age≤40	2.237 (1.381-3.623)	0.001
constant	0.177	<0.001
Factors correlated with single polysomy		
Higher tumor grade	1.740 (1.185-2.556)	0.005
Ki-67≥20%	2.024 (1.062-3.857)	0.032
constant	0.022	<0.001

Univariate analysis revealed that age (*P*=0.041), tumor grade (*P*=0.001) and Ki-67 (*P*=0.002) were correlated with 1q/19p single polysomy. Logistic regression analysis confirmed higher tumor grade (OR 1.740, 95%CI 1.185-2.556, *P*=0.005) and Ki-67 ≥20% (OR 2.024, 95%CI 1.062-3.857, *P*=0.032) as independent factors correlated with 1q/19p single polysomy (Table 3).

### 1q/19p co-polysomy predicts longer survival whereas single polysomy predicts shorter survival in astrocytic gliomas

**Figure 1:**
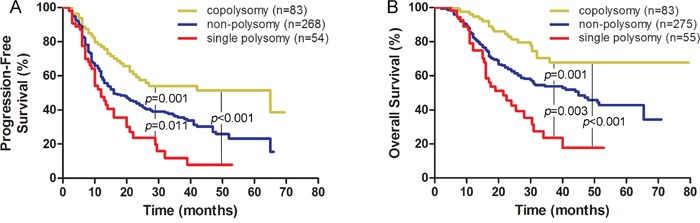
1q/19p co-polysomy predicted longer survival, whereas single polysomy predicted shorter survival in the WHO_2007_ classified cohort (**A** for PFS and **B** for OS).

Of 421 patients, 85 harbored the 1q/19p co-polysomy, 58 harbored the 1q/19p single polysomy, and 278 harbored no polysomy. Of the three subgroups, patients with 1q/19p co-polysomy had the longest progression-free survival (PFS) and overall survival, patients with 1q/19p single polysomy had the shortest PFS and OS, and patients without polysomy had intermediate PFS and OS (Figure 1A and 1B). The median PFS of three subgroups were 65.0 months, 16.0 months and 12.0 months, respectively. The median OS of the three subgroups were N/A, 45.0 months and 21.5 months, respectively.

Patients with co-polysomy had longer PFS and OS than did those without co-polysomy (PFS: 65.0 months vs. 16.0 months, *P*<0.001; OS: N/A vs. 34.0 months, *P*<0.001, Supplementary Figure 1A and 1B).

Patients with single polysomy had shorter survival than did those without single polysomy (PFS: 12.0 months vs. 21.0 months, *P*=0.001; OS: 21.5 months vs. 51.0 months, *P*<0.001, Supplementary Figure 1C and 1D).

### 1q/19p co-polysomy predicts longer survival in the A, AA and GBM subgroups

Subgroup analysis confirmed that 1q/19p co-polysomy predicted longer PFS and OS in the A, AA, and GBM subgroups. In the subgroup with astrocytomas (A, WHO grade II), the median PFS for patients with and without co-polysomy were N/A and 42.0 months (*P*=0.013, Supplementary Figure 2A), and the median OS were N/A and 65.5 months, respectively (*P*=0.047, Supplementary Figure 2B). In the subgroup with anaplastic astrocytomas (AA, WHO grade III), the median PFS for patients with and without co-polysomy were 26.0 and 12.0 months (*P*=0.018, Supplementary Figure 2C), and the median OS were N/A and 24.0 months, respectively (*P*=0.008, Supplementary Figure 2D). In the subgroup with glioblastoma (GBM, WHO grade IV), the median PFS for patients with and without co-polysomy was 14.0 and 10.0 months (*P*=0.047, Supplementary Figure 2E), and the median OS were 32.0 and 18.5 months, respectively (*P*=0.019, Supplementary Figure 2F).

### 1q/19p single polysomy predicts shorter survival in the GBM subgroup

**Figure 2:**
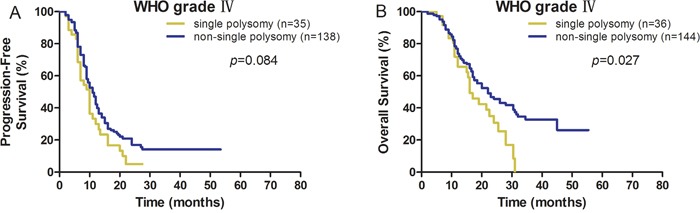
1q/19p single polysomy predicted shorter survival in GBM (**A** for PFS and **B** for OS).

Subgroup analysis confirmed that 1q/19p single polysomy predicted longer PFS and OS in the GBM subgroup but not in the A and AA subgroups due to limited cases. In the GBM subgroup, the median PFS for patients with and without single polysomy were 10.0 and 11.0 months (*P*=0.084, Figure 2A). The median OS for patients with and without co-polysomy were 16.0 and 22.0 months (*P*=0.027, Figure 2B)

### Factors correlated with survival in astrocytic gliomas by log-rank analysis

The prognostic factors associated with PFS and OS were analyzed by Kaplan-Meier survival analyses (Table 4). According to the log-rank analysis, the clinical factors correlated with longer PFS included age ≤40 (*P*<0.001), KPS ≥70 (*P*<0.001), total resection of tumor (*P*=0.034), tumor size <6 cm (*P*=0.041), Ki-67 <20% (*P*<0.001), lower tumor grade (*P*<0.001), 1q/19p co-polysomy (*P*<0.001), no single polysomy (*P*=0.001), and IDH1/2 mutation (*P*=0.007), as shown in Table 4. The factors correlated with longer OS included age ≤40 (*P*<0.001), KPS ≥70 (*P*=0.006), total resection of tumor (*P*=0.001), tumor size <6 cm (*P*=0.034), Ki-67 <20% (*P*<0.001), lower tumor grade (*P*<0.001), 1q/19p co-polysomy (*P*<0.001), no single polysomy (*P*<0.001), and IDH1/2 mutation (*P*=0.001).

**Table 4:** Log rank analysis of clinical factors correlated with survivals of the WHO_2007_ cohort

Clinical factors	Median PFS in months (95% CI)	No. of patients*	*P* value	Median OS in months (95% CI)	No. of patients*	*P* value
Age (yrs)						
≤40	41.0 (28.0-54.0)	157	**<0.001**	N/A	159	**<0.001**
>40	13.0 (11.5-14.5)	248		27.0 (21.3-32.7)	254	
Gender						
Male	19.5 (14.9-24.1)	243	0.824	45.0 (32.9-57.1)	248	0.644
female	20.0 (13.8-26.0)	162		36.0 (20.3-51.7)	165	
KPS						
≥70	21.0 (15.8-26.2)	229	**<0.001**	N/A	232	**0.006**
<70	12.0 (9.0-15.0)	55		24.0 (14.7-33.3)	58	
Removal degree						
GTR	20.0 (12.0-22.0)	268	**0.034**	N/A	272	**0.001**
Non-GTR	17.0 (13.8-26.1)	137		30.5 (25.9-35.1)	141	
Tumor size						
≥ 6 cm	15.0 (10.0-20.0)	160	**0.041**	32.0 (22.7-41.3)	164	**0.034**
< 6 cm	21.0 (15.7-26.3)	245		N/A	249	
Ki-67						
<20%	23.0 (18.2-27.8)	296	**<0.001**	N/A	299	**<0.001**
≥20%	11.0 (8.6-13.4)	77		22.0 (16.1-27.9)	81	
Tumor grade						
WHO II	48.0 (34.9-61.1)	155	**<0.001**	N/A	155	**<0.001**
WHO III	21.0 (13.3-28.6)	77		30.0 (20.2-39.8)	78	
WHO IV	10.0 (8.5-11.5)	173		21.5 (17.5-25.5)	180	
1q/19p co-polysomy						
Yes	65.0 (11.2-118.8)	83	**<0.001**	N/A	83	**<0.001**
No	16.0(12.4-19.6)	322		34.0 (24.2-43.8)	330	
single polysomy						
Yes	12.0 (9.1-14.9)	54	**0.001**	21.5 (13.9-29.1)	55	**<0.001**
No	21.0 (16.1-25.9)	351		51.0 (37.1-64.9)	358	
IDH1/2 mutation						
Yes	28.0 (20.5-35.5)	120	**0.007**	51.5 (N/A)	123	**0.001**
No	18.0 (13.3-22.7)	195		25.0 (23.3-44.7)	199	

*PFS was not available in 16 cases and OS was no available in 8 cases. GTR=gross-total resection.

### 1q/19p co-polysomy independently predicts longer survival in astrocytic gliomas by Cox regression

Age, KPS, tumor resection degree, tumor size, Ki-67 index, tumor grade, 1q/19p co-polysomy, 1q/19p single polysomy, and IDH1/2 mutation were included in Cox regression analysis (Table 5). In the Cox regression model, the factors independently correlated with PFS were 1q/19p co-polysomy (OR 0.538, 95%CI [0.374-0.772], *P*=0.001), age ≤40 (OR 0.715, 95% CI [0.536-0.955], *P*=0.023), higher tumor grade (OR 2.124, 95%CI [1.808-2.495], *P*<0.001), and gross-total resection of tumor (OR 0.592, 95%CI [0.455-0.772], *P*<0.001). The factors independently correlated with OS were 1q/19p co-polysomy (OR 0.460, 95%CI [0.287-0.738], *P*=0.001), age ≤40 (OR 0.627, 95%CI [0.428-0.918], *P*=0.017), higher tumor grade (OR 2.250, 95%CI [1.843-2.747], *P*<0.001), and gross-total resection of tumor (OR 0.461, 95%CI [0.337-0.630], *P*<0.001).

**Table 5:** Cox regression model in association with prognoses in the WHO_2007_ cohort (N=421)

Factors	PFS		OS	
	OR (95% CI)	*P* value	OR (95% CI)	*P* value
1q/19p co-polysomy (yes/no)	0.538 (0.374-0.772)	0.001	0.460 (0.287-0.738)	0.001
Age (≤40/>40)	0.715 (0.536-0.955)	0.023	0.627 (0.428-0.918)	0.017
Tumor grade (IV/III/II)	2.124 (1.808-2.495)	<0.001	2.250 (1.843-2.747)	<0.001
Removal degree (GTR/non-GTR)	0.592 (0.455-0.772)	<0.001	0.461 (0.337-0.630)	<0.001

PFS=progression-free survival; OS=overall survival; OR=odd ratio; CI=confidence interval. GTR=gross-total resection.

### Detection of chromosome 8 polysomy in gliomas

**Figure 3:**
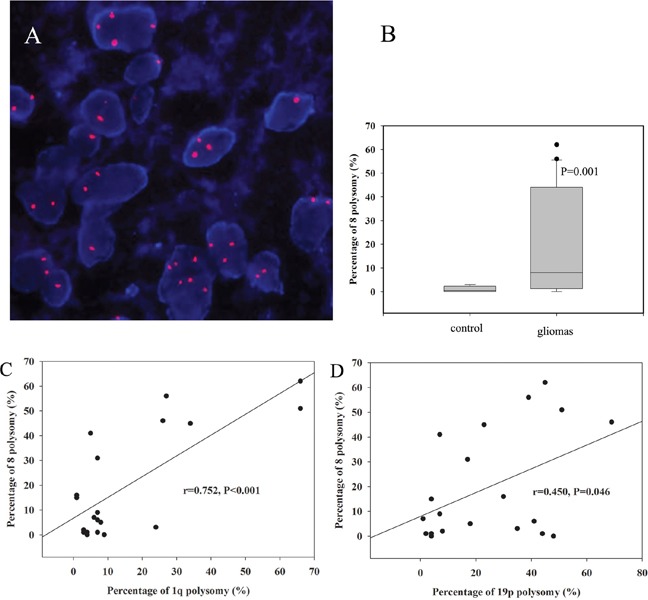
Chromosome 8 polysomy was shown in gliomas, including trisomy, tetrasomy and polysomy of more than 5 copies **(A)**. The percentage of chromosome 8 polysomy in gliomas was higher than that in the control **(B)**. The percentage of chromosome 8 polysomy was correlated with 1q polysomy **(C)** and 19p polysomy **(D)**.

To investigate the co-occurrence of polyploidy and polysomy, we investigated chromosome 8 polysomy in 20 glioma specimens by CEP8-FISH. Ten tumor cell-free surrounding tissues evidenced under pathological microscopy were selected from 10 patients as negative controls. Chromosome 8 polysomy could be found in gliomas, including trisomy, tetrasomy and polysomy of more than 5 copies (Figure 3A). The percentages of chromosome 8 polysomy in gliomas were higher than those in the negative control (*P*=0.001, Figure 3B). The percentage of chromosome 8 polysomy was correlated with 1q polysomy (r=0.752, *P*<0.001, Figure 3C) and 19p polysomy (r=0.450, *P*=0.046, Figure 3D). The results showed that chromosome 8 polysomy co-existed with the 1q/19p polysomy in these 20 glioma specimens, which indicated the polyploidy of the tumor.

### Validation using the WHO_2016_ classified cohort of astrocytic tumors

**Figure 4:**
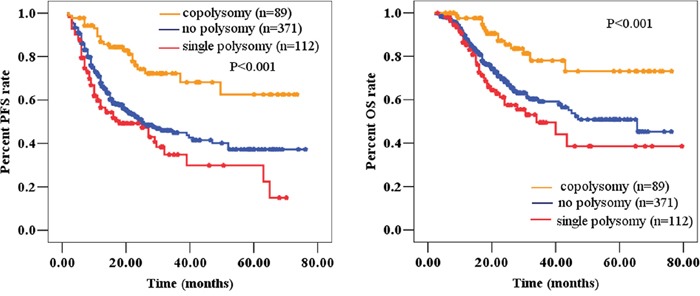
1q/19p co-polysomy predicted longer survival, whereas single polysomy predicted shorter survival (**A** for PFS and **B** for OS) in the WHO_2016_ classified cohort.

To validate the independent survival impact of the 1q/19p co-polysomy, we assessed the WHO_2016_ cohort (N=572, Supplementary Table 1). In Cox regression models, we found that 1q/19p co-polysomy was also an independent factor correlated with prognosis in astrocytic gliomas after controlling for age, grade, IDH1/2 mutation, and removal degree (*P*<0.05, Figure 4 and Table 6).

**Table 6:** Cox regression model in association with prognoses in the WHO_2016_ cohort (n=572)

Factors	PFS		OS	
	OR (95% CI)	*P* value	OR (95% CI)	*P* value
1q/19p co-polysomy (yes/no)	0.503 (0.322-0.785)	0.003	0.557 (0.318-0.976)	0.041
Age (≤40/>40)	0.589 (0.443-0.784)	<0.001	0.565 (0.386-0.826)	0.003
Tumor grade (IV/III/II)	2.279 (1.907-2.724)	<0.001	2.266 (1.820-2.822)	<0.001
Removal degree (GTR/non-GTR)	0.667 (0.509-0.875)	0.003	0.481 (0.346-0.668)	<0.001
IDH1/2 mutation (yes/no)	0.782 (0.580-1.055)	0.107	0.638 (0.440-0.924)	0.018

PFS=progression-free survival; OS=overall survival; OR=odd ratio; CI=confidence interval. GTR=gross-total resection.

## DISCUSSION

According to the histological findings, gliomas can be divided into astrocytic, oligodendroglial, and mixed tumors. Compared with oligodendroglial and mixed tumors, astrocytic tumors exhibited worse prognoses. Recently, molecular biomarkers including the 1p/19q co-deletion, BRAF mutation, IDH1/2 mutation, and TERT mutation have been widely used in the diagnosis, treatment, and prognostic prediction of gliomas. The 1q/19p polysomy predicts unfavorable prognoses in the setting of 1p/19q co-deleted oligodendroglial tumors (ODGs) [7–9]. In our previous study, we confirmed the prognostic merit of 1q/19p co-polysomy in 148 1p/19q co-deleted ODGs [9]. However, the prognostic merit of 1q/19p co-polysomy was unknown in astrocytic gliomas. Now, in the present study, we analyzed the prognostic merit of 1q/19p co-polysomy in the WHO_2007_ and WHO_2016_ classified cohorts of astrocytic gliomas with longer follow-up periods.

So far, this is the first study to focus on 1q/19p co-polysomy in astrocytic tumors. For the first time, we found that 1q/19p co-polysomy predicted longer survival in astrocytic tumors, irrespective of tumor grade. This finding was validated by the WHO_2016_ classified cohort. Geisenberger identified the 19 and 20 co-polysomy as a favorable prognostic marker in glioblastomas [11]. Previous studies regarding ploidy as a prognostic marker in gliomas were contradictory. Some studies found that the percentages of DNA aneuploidy in gliomas correlated with tumor grade and shorter survival [12–16], whereas others found no association [17–19], and still others reported associations with longer survival [20–22]. El-Rayes et al. implied that cellular DNA content parameters may correlate with the natural history and treatment outcomes of newly diagnosed untreated patients with astrocytomas [23].

The prognostic merit of co-polysomy in astrocytic tumors was opposite to that in oligodendroglial tumors. This may explain why previous studies about ploidy as a prognostic marker in gliomas were contradictory. To clarify the prognostic merit of polyploidy or polysomy in gliomas, detailed stratification is necessary. In this study, 1q/19p co-polysomy predicted longer survival, whereas single polysomy predicted shorter survival in astrocytic tumors. In oligodendroglial tumors, however, patients with co-polysomy had worse prognoses regardless of tumor grade, especially in the 1p/19q co-deleted subgroups [7–10]. Perhaps this could be explained by the theory of the aneuploidy paradox [24]. The beneficial effects of aneuploidy in enhancing cell growth will be most evident under stringent selective pressures and induce a “mutator phenotype” that increases DNA damage and genomic instability [24].

To clarify the relationship between polyploidy and polysomy in gliomas, we assessed the frequency of chromosome 8 polysomy in gliomas. We found that chromosome 8 polysomy was correlated with 1q polysomy and 19p polysomy. The correlation of two independent polysomy implied tumor polyploidy [25].

To further identify the mechanism between co-polysomy and favorable prognoses, we analyzed the factors correlated with co-polysomy. Age ≤40 was correlated with 1q/19p co-polysomy as an independent factor. Ki-67 was not correlated with co-polysomy in astrocytic tumors. 1q/19p co-polysomy correlated with younger age, which was consistent with the results of Andrea L’s report [8]. It was also reported that younger patients were strongly correlated with longer overall survivals in gliomas [19, 20, 26, 27]. In addition, Perry reported that aneuploidy in anaplastic astrocytic tumors was associated with younger age and longer survival, whereas diploidy was associated with older age and shorter survival [20]. It was also speculated that radiotherapeutic and chemotherapeutic treatments may be more effective in aneuploid tumors and account for improved survival [20].

For the first time, we reported that the 1q/19p single polysomy predicted the shortest survival in astrocytic gliomas, although it was not confirmed as an independent factor in Cox regression. The median PFS and OS were 12.0 months and 21.5 months, respectively, in the WHO_2007_ classified cohort. Subgroup analysis confirmed this trend in GBM. This finding was also validated by the WHO_2016_ classified cohort. To further identify the mechanism between single polysomy and unfavorable prognoses, we analyzed the factors correlated with single polysomy. Higher tumor grade and Ki-67 ≥20% were independent factors correlated with single polysomy. It was speculated that single polysomy was associated with greater malignancy and proliferative activity.

### Study limitations

The greatest limitation of this study is the lack of validation of polysomy by other methods. For the first time, Snuderl et al. provided the definition of polysomy of 1 and 19 based on the FISH by-product result in the detection of 1p/19q co-deletion [7]. Strictly speaking, polysomy is a copy number gain of an entire body of a specific chromosome. An increased number of probe signals indicated local amplification of the locus targeted by FISH probes. These results were not validated using other methods, such as array CGH or MLPA.

This is a retrospective study involving cases from 2009 to 2016, and biomarkers such as MGMT, TERT, ATRX, and H3K27 were not available for all cases and were not analyzed in this article. In addition, many tumors showed high variability in copy number, which precluded our further sub-stratification on the copy number.

In conclusion, we reported the prognostic significance of the 1q/19p polysomy in astrocytic tumors. 1q/19p co-polysomy independently predicted longer survival after adjusting for the commonly applied standard prognostic markers. Therefore, it could be useful in the molecular stratification of gliomas.

## MATERIALS AND METHODS

### Ethics statement

A WHO_2007_ classified cohort of patients (N=421) with astrocytic glioma was enrolled in the study. A WHO_2016_ cohort (N=572) with astrocytic gliomas was used as a validated cohort. All patients provided written informed consent for the current study, and the clinical study was approved by the Medical Ethics Committee of Capital Medical University.

### Pathological examination

For the WHO_2007_ classified cohort (N=421), all specimens were independently reviewed and graded by two senior neuropathologists according to the 2007 WHO Classification of Tumors of the Central Nervous System [28]. For the WHO_2016_ cohort (N=572), assessments were made according to the 2016 WHO Classification of Tumors of the Central Nervous System [6]. The histological diagnoses of the tumor specimens were reviewed and confirmed by a third senior neuropathologist. If the first two pathologists did not agree on the diagnosis, a third senior neuropathologist would resolve the judgment. If the three neuropathologists could not reach an agreement, the case was submitted to the pathological committee of Beijing Neurosurgical Institute and Beijing Tiantan Hospital for final diagnosis.

### Recording of clinical material

The clinical, radiological, operative, and pathological records were recorded. All of the patients were closely followed including records of adjuvant therapies, neuro-imaging, PFS and OS. Tumor size was defined as the maximal diameter of the tumor in the axial, sagittal or coronary planes on enhanced T1 images for high-grade gliomas and on T2/Flair images for low-grade gliomas. Tumor resection degree was defined according to the reported criteria [29–31]. The PFS was defined as the duration from the date of surgery to the date of recurrence as demonstrated by radiology. The OS was defined as the duration from the date of surgery to the date of death. The KPS score was used to judge preoperative functional status. Peri-operative death, which was defined as death within 30 days of surgery, was excluded from this study.

### Detection of the 1p/19q co-deletion and 1q/19p polysomy by FISH method

**Figure 5:**
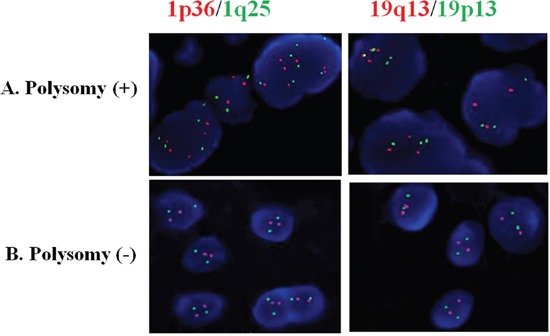
Astrocytic tumor with 1q/19p co-polysomy **(A)** and without polysomy **(B)** by FISH detection. The red signal represents the 1p36 and 19q13 FISH probes, whereas the green signal represents the 1q25 and 19p13 FISH probes.

The 1p/19q fluorescent probe kit (Vysis, USA) was used for the FISH test as was described previously [9, 32]. The assessment and interpretation of FISH results were performed according to guidelines defined by the SIOP Europe Neuroblastoma Pathology and Biology and Bone Marrow Group [33]. Tumors with more than 30% of nuclei showing DNA loss were defined as tumors with chromosomal loss. The tumor was considered to have a 1q and 19p polysomy if 30% of nuclei showed more than two 1q or 19p deletions [7] (Figure 5). All cases with 1p/19q co-deletions were excluded.

### Quality control for FISH detection of 1p/19q co-deletion and polysomy

For each case, a paraffin-embedded tumor block was selected based on tumor content, including the highest grade component and representation of the predominant morphology of the individual case. Several sections were prepared for FISH. The first and last sections were hematoxylin and eosin stained, and regions representing tumor were delineated. The first section was examined to ensure that it met the standards by which the block was selected. For FISH analysis, the section immediately adjacent to the first hematoxylin and eosin stained slide was used to minimize the effects of tumor heterogeneity. In the corresponding region rich in tumor cells, more than 100 non-overlapping nuclei were enumerated per hybridization for each probe. Some parameters were used for quality control as reported previously [9].

### IDH1/2 sequence analysis

Genomic DNA was isolated from snap-frozen tissues using the QIAmp DNA mini-kit, as instructed by the manufacturer (Qiagen). A fragment 254 bp in length spanning the catalytic domain of IDH1 and including codon 132 was amplified using the sense primer IDH1 F: 5’-ACCAAATGGCACCATACG-3’ and the antisense primer IDH1 R: 5’-TTCATACCTTGCTTAATGGGG-3’. A fragment 293 bp in length spanning the catalytic domain of IDH2 and including codon 172 was amplified using the sense primer IDH2 F: 5’-GCTGCAGTGGGACCACTATT-3’ and the antisense primer IDH2 R: 5’-TGTGGCCTTGTACTGCAGAG′. PCR using standard buffer conditions, 30 ng of DNA and GoTaq DNA Polymerase (TaKaRa, Japan) employed 35 cycles with denaturing at 95°C for 30 s, annealing at 54°C for 45 s and extension at 72°C for 50 s in a total volume of 25 μL. The PCR amplification product was then sequenced for analysis.

### Assessment of chromosome 8 polysomy in glioma specimens by FISH

Chromosome 8 polysomy was detected in 20 patient tumor specimens by FISH, and tumor cell-free surrounding tissues evidenced under pathologic microscopy were selected from 10 patients as negative controls. The Vysis CEP8 SpectrumOrange Direct Labeled Fluorescent DNA Probe kit was used for FISH testing. Paraffin slides (4 μm thick) were deparaffinized, dehydrated, and incubated in 1 mol/L NaSCN for 35 min at 80°C. The slides were then immersed in pepsin solution (0.65 % in protease buffer with 0.01 mol/L HCl) for 10 min at 37°C, and the tissues were fixed in 10 % neutral buffered formalin. The specimens were then dehydrated in ethanol (70, 85, and 100 %, 2 min in each bath) and air-dried. Twenty microliters of each probe was then added separately, and the slides were sealed with rubber cement. After co-denaturation for 10 min at 75°C, the slides were then placed in a humidified atmosphere with Hybrite (ThermoBriteTM vysis) for 16 h at 37°C. The slides were then immersed in 2x SSC/0.3 %NP-40 for 2 min at RT and then in 2xSSC/0.3 % NP-40 for 2 min at 73°C. After drying, the nuclei were counterstained with 4,6-diamidino2-phenylindole (DAPI) and an antifade compound (p-Phenylenediamine). FISH signals for each locus-specific FISH probe were assessed using an Olympus BX51TRF microscope (Olympus, Ina-shi, Nagano, Japan) equipped with a triple-pass filter (DAPI/Green/Orange; Vysis). The entire areas of the tissue microarray cores were evaluated in each case, and as many non-overlapping nuclei as possible (≥100 per hybridization) were assessed for red (target) signals.

### Statistics

Summary data are presented as the mean ± SD for parametric data and as the median with the IQR in parentheses for nonparametric data. For intergroup comparisons, Student’s t-test was used for parametric data and the Mann-Whitney U-test for nonparametric data. Percentages were compared using the chi-square test or Fisher’s exact test where appropriate. Survival as a function of time was plotted using the Kaplan-Meier method, and the log-rank analysis was used to compare Kaplan-Meier plots. Multivariate proportional hazards regression analysis was used to identify factors associated with PFS and OS. In this analysis, all variables associated with survival in univariate analysis (*P*<0.05) were included in a step-wise multivariate proportional hazards regression model. SPSS 13.0 (SPSS for Windows, version 13.0 [SPSS Inc., Chicago, Illinois, USA]) was used for statistical analysis. Probability values were obtained using 2-sided tests with statistical significance defined as *P*<0.05.

## SUPPLEMENTARY MATERIALS FIGURES AND TABLE



## Abbreviations

WHO: World Health Organization
FISH: fluorescence *in situ* hybridization
GTR: gross total resection
A: astrocytoma
AA: anaplastic astrocytoma
GBM: glioblastoma
PFS: progression-free survival
OS: overall survival.
