# Supplementary material for: 1q/19p co-polysomy predicts longer survival in patients with astrocytic gliomas
Source: Oncotarget. 2017 May 16;8(40):67104–16. doi: 10.18632/oncotarget.17947 (PMC5620159; doi:10.18632/oncotarget.17947)
Supplement: Supplementary file 1 [file oncotarget-08-67104-s001.pdf]

1q/19p co-polysomy predicts longer survival in patients with astrocytic gliomas

SUPPLEMENTARY MATERIALS

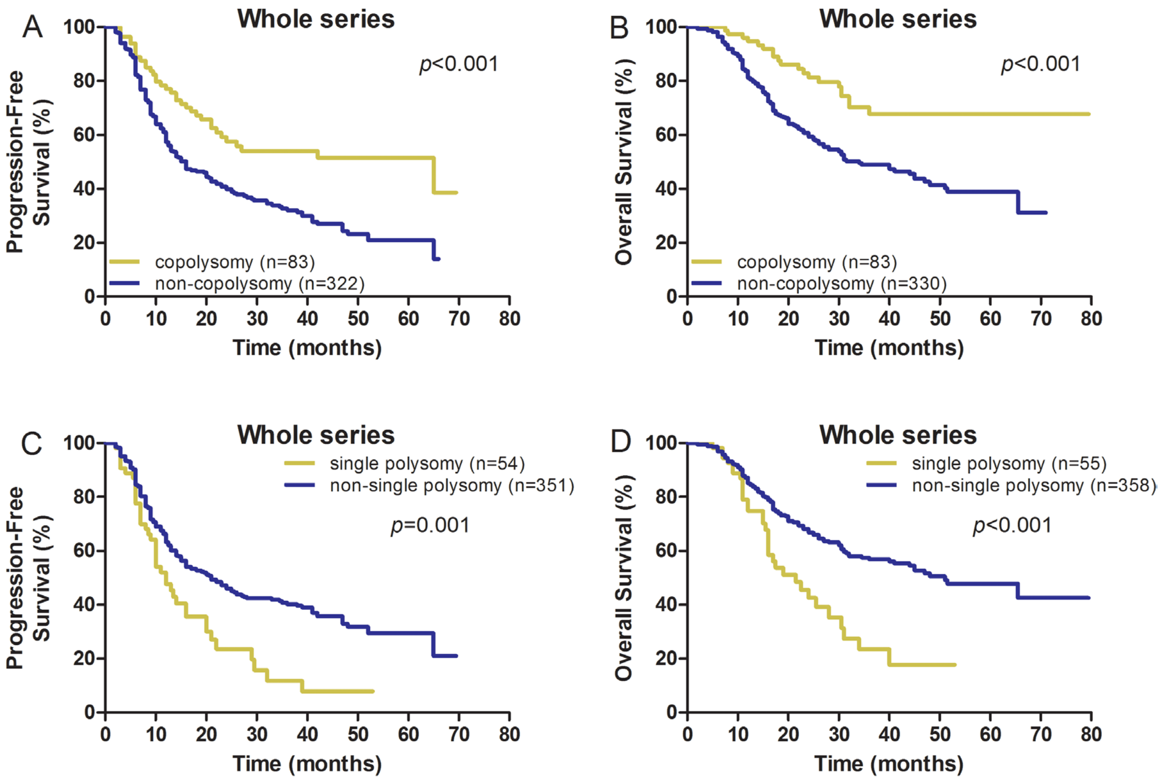

Supplementary Figure 1: Patients with 1q/19p co-polysomy had longer survival than those without co-polysomy (**A** for PFS and **B** for OS). Patients with the 1q/19p single polysomy had shorter survival than did those without single polysomy (**C** for PFS and **D** for OS).

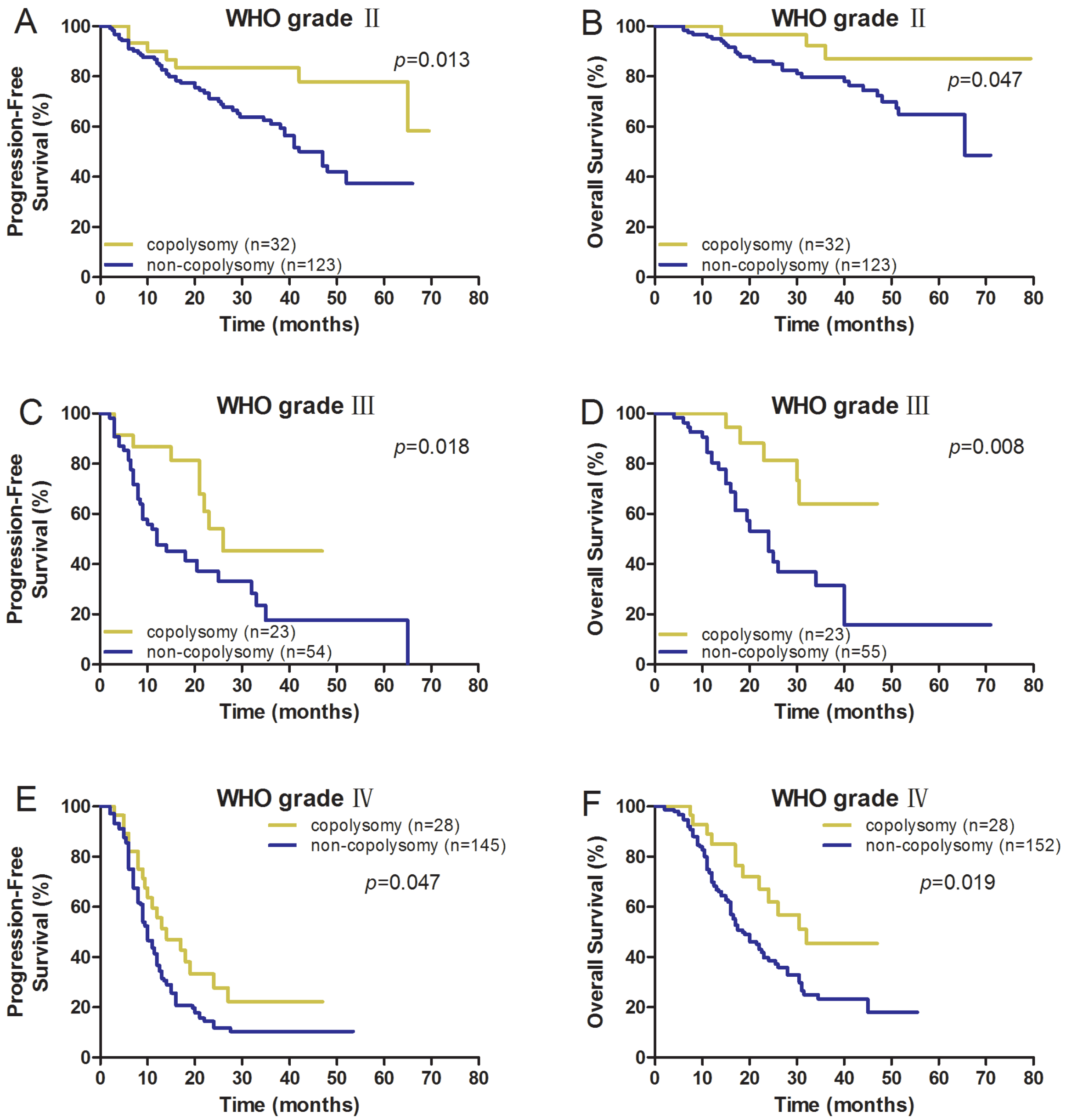

Supplementary Figure 2: 1q/19p co-polysomy predicted longer survival in grade II (**A** for PFS and **B** for OS), grade III **(C** for PFS and **D** for OS), and grade IV gliomas **(E** for PFS and **F** for OS).

Supplementary Table 1: Pathological classification in the WHO_{2016} classified cohort (n=572)

Classification	Number
Diffuse astrocytoma, IDH-mutant	158
Diffuse astrocytoma, IDH-wildtype	79
Anaplastic astrocytoma, IDH-mutant	70
Anaplastic astrocytoma, IDH-wildtype	66
Glioblastoma, IDH-mutant	40
Glioblastoma, IDH-wildtype	159
